# Supplementary material for: Ammonia and Nematode Ascaroside Are Synergistic in Trap Formation in Arthrobotrys oligospora
Source: Pathogens. 2023 Aug 31;12(9):1114. doi: 10.3390/pathogens12091114 (PMC10536950; doi:10.3390/pathogens12091114)
Supplement: Supplementary file 1 [file pathogens-12-01114-s001.zip › pathogens-2555357-supplementary.pdf]

pathogens

MDPI

Supplementary Materials:

Table S1. List of primers in this study.

Primers	Sequence (5′-3′)	Description
Amt43-5F	GTAACGCCAGGGTTTTCCCAGTCACGACGCTCGTTAGGACCAGCACTATGT	*AoAmt43* 5′flank
Amt43-5R	ATCCACTTAACGTTACTGAAATCTCCAACACCAACCATCAAGGCGTAGG	
Amt43-3F	CTCCTTCAATATCATCTTCTGTCTCCGACTCACTGTCACCTGTCTCATTCT	*AoAmt43* 3′flank
Amt43-3R	GCGGATAACAATTTCACACAGGAAACAGCCGAACACGAGCACATACATAGA	
Hph-F	GTCGGAGACAGAAGATGATATTGAAGGAGC	HPH
Hph-R	GTTGGAGATTTCAGTAACGTTAAGTGGAT	
*Amt43ko*_F	CTCGGGACAATCTTCCTCTG	Probe of Southern blot
*Amt43ko*_R	CGTATGCGAGGATAGCAACA	
Amt43P-F	GCTCTCCGTAAGGATGCTTCGCAGGTATCGCGCATATAC	Promoter
Amt43P-R	CTCCTCGCCCTTGCTCACCATAGTTAGGTATAAGTCGGTGAC	
Amt43-F	GTCACCGACTTATACCTAACTATGTCGGCTGAAGAGCCAAT	*Amt43*
Amt43-R	CCTCGCCCTTGCTCACCATCTACGGTTTCGCTTCTACCTGA	
Amt43T-F	TCACGGCATGGACGAGCTGTACAAGTAAAATGATCGTCTTCTGCTTTGG	terminator
Amt43T-R	ATCCCCCGGGCTGCAGGAATTCCTGTATCTTGCAGAATGG	
*Amt43*Nat-F	TTGGAGCCGAAATCCGCATGGACATGGAGGCCCAGAATAC	Norsmectin fragment
*Amt43*Nat--R	AAGTCCGGCACCTCGTGCAGTATAGCGACCAGCATTC	

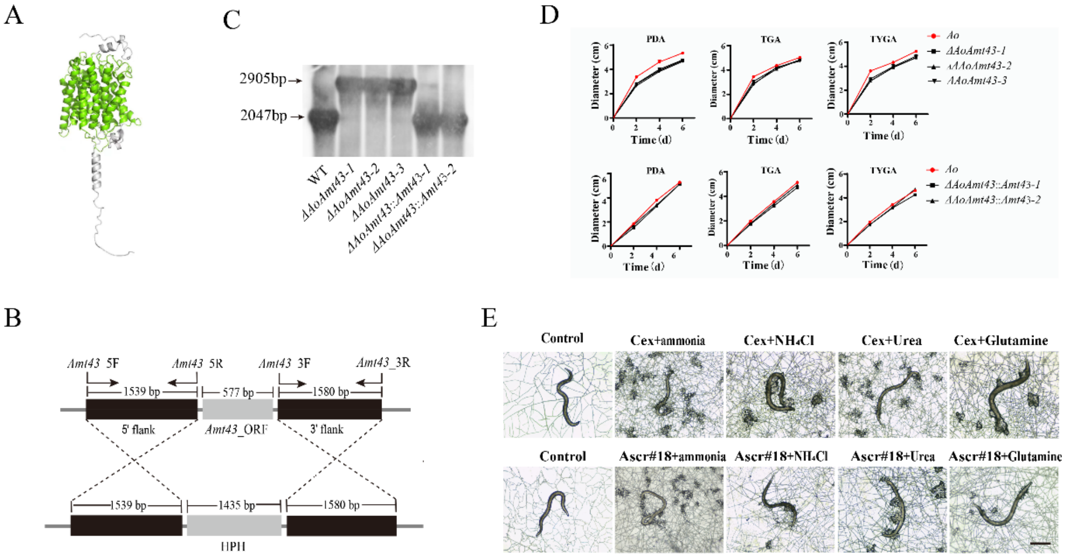

Figure S1. Protein structure analysis of Amt43 and its knockout. (A) Protein structure of Amt43; (B) Amt43 knockout programme. (C) Southern blot validation of Δ*AoAmt43* and Δ*AoAmt43:: Amt43*. (D) Mycelial growth in the WT, Δ*AoAmt43* and Δ*AoAmt43:: Amt43* on PDA, TGA, or TYGA media for 6 days. (E) Comparison of nematode pathogenicity of traps induced by nematode extracts and nitrogen signalling mixtures against nematodes,and comparison of nematode pathogenicity of traps induced by Ascr#18 and nitrogen signalling mixtures against nematodes (Bar=100 μm).
